# Uptake and Short-term Outcomes of High-risk Screening Colonoscopy Billing Codes: A Population-based Study Among Young Adults

**DOI:** 10.1093/jcag/gwab014

**Published:** 2021-06-10

**Authors:** Lawrence Paszat, Rinku Sutradhar, Jin Luo, Jill Tinmouth, Linda Rabeneck, Nancy N Baxter

**Affiliations:** Dalla Lana School of Public Health, University of Toronto, Toronto, Ontario, Canada; Dalla Lana School of Public Health, University of Toronto, Toronto, Ontario, Canada; Cancer Research Programme, Institute for Clinical Evaluative Sciences (ICES), Toronto, Ontario, Canada; Dalla Lana School of Public Health, University of Toronto, Toronto, Ontario, Canada; Dalla Lana School of Public Health, University of Toronto, Toronto, Ontario, Canada; School of Population and Global Health, University of Melbourne, Melbourne, Victoria, Australia

**Keywords:** Colorectal cancer family history, Hereditary colorectal cancer syndromes, Mean cumulative function, Screening colonoscopy, Surveillance colonoscopy

## Abstract

**Background:**

Persons suspected or confirmed with familial colorectal cancer syndrome are recommended to have biennial colonoscopy from late adolescence or early adulthood. Persons without a syndrome but with one or more affected first-degree relatives are recommended to begin colonoscopy 10 years before the age at diagnosis of the youngest affected relative, and every 5 to 10 years. Ontario introduced colonoscopy billing codes for these two indications in 2011.

**Methods:**

We identified persons in Ontario under 50 years of age, without a prior history of colorectal cancer or inflammatory bowel disease, with one or more of these billing claims between 2013 and 2017. We described the index colonoscopy, and subsequent colonoscopy up-to-date status. We computed average annual rates of colorectal and other cancer diagnoses, and displayed mean cumulative function plots, stratified by billing code, age and sex.

**Results:**

Billing claims for ‘familial syndrome’ high-risk screening colonoscopy were identified among 14,846 persons; the average annual rate of CRC diagnoses was 38.6 per 100,000 among males and 22.2 among females. Colonoscopy up-to-date status fell to 50% within 7 years. Billing claims for ‘first-degree relative’ screening colonoscopy was identified among 49,505 persons; average annual rates of CRC diagnoses were 16.3 among males and 13.5 per 100,000 among females, respectively.

**Conclusion:**

Colorectal cancer was more frequent following billing claims for high-risk screening colonoscopy for familial syndromes, as were noncolorectal malignancies potentially associated with these syndromes. This billing claim for familial colorectal cancer syndrome colonoscopy appears to identify a group at elevated short-term risk for cancer.

## Introduction

Persons suspected or confirmed to have a familial colorectal cancer syndrome are recommended to have biennial colonoscopy from late adolescence or early adulthood (1–3). Persons without a syndrome but with one or more affected first-degree relatives are recommended to begin colonoscopy at least 10 years before the age at diagnosis of the youngest affected relative, and every 5 to 10 years (1–3).

Physicians and surgeons in Ontario are reimbursed for colonoscopy by the Ontario Health Insurance Plan (OHIP) (4). Until recently, there was one feecode for colonoscopy for all indications (Z555 to the descending colon, with additional codes for each proximal segment of the colon examined). Ten additional fee codes for colonoscopy, for various indications, were introduced between 2011 and 2013. Notably, Z494 was introduced for diagnosis or ongoing management of ‘Hereditary (e.g., Familial Adenomatous Polyposis or Hereditary Non-Polyposis Colorectal Cancer or other bowel disorders associated (e.g., inflammatory bowel disease) with increased risk of malignancy’, and Z499 was introduced for colonoscopy in the ‘absence of signs or symptoms, with family history associated with an increased risk of malignancy (e.g., a first-degree relative or at least two second-degree relatives with colorectal cancer or a premalignant lesion)’.

Ontario funds genetic testing for Lynch Syndrome (5) in addition to colonoscopy. However, studies from several countries demonstrate that members of families, among whom a mutation has been identified, are often unwilling to undergo genetic testing or frequent colonoscopy (6,7).

This work focuses on permanent residents of Ontario age < 50 years on the date of their first Z494 or Z499 billing claim for colonoscopy between 2013 and 2017, who are not found in the Ontario Crohn’s and Colitis Database at any time. There are recommendations for colonoscopic screening among those in this age group with familial syndromes or other family history (1–3), and among whom the incidence of colorectal cancer is rising regardless of family history (8–10). This work will be the first examination of these feecodes and the previous history and/or subsequent health events consistent with elevated familial risk of colorectal cancer among persons with one or more of these records.

## METHODS

We identified the first billing claim for OHIP feecode Z494 (Colonoscopy for Hereditary [e.g., Familial Adenomatous Polyposis or Hereditary Non-Polyposis Colorectal Cancer] or other bowel disorders [e.g., inflammatory bowel diseases] associated with increased risk of malignancy) among persons aged <= 49 years between 2013 and 2017 (4). Persons with any record of colorectal cancer (CRC, International Classification of Diseases version 10 (ICD-10) C18^ - C20^) in the Ontario Cancer Registry prior to the date of the Z494 billing claim (the index date) were excluded, as were any persons present in the Ontario Crohn’s and Colitis Cohort (OCCC) database at any time before or after the index date.

Among persons aged <= 49 years without a billing claim for OHIP feecode Z494, we identified the first billing claim for OHIP feecode Z499 (colonoscopy for persons in the absence of signs or symptoms with family history associated with an increased risk of malignancy, e.g., a first-degree relative or at least two second-degree relatives with colorectal cancer or a premalignant lesion) for a patient 40 years of age or older, or, 10 years younger than the earliest age of diagnosis of the youngest affected relative) (4), and excluded those with prior CRC or with a record in the OCCC at any time. These datasets were linked using unique encoded identifiers and analyzed at ICES.

Prior to the index date, we computed the percent of persons who were ‘up-to-date’ with colonoscopy at the end of each year. We computed the percent of persons with one or more billing claims for polypectomy <=5 years, and > 5 years to 10 years prior to the index date. From the Ontario Cancer Registry we extracted records of cancers potentially associated with hereditary CRC syndromes with diagnosis dates prior to the index date (ICD-10: [International Classification of Diseases version 10] C16^ - C17^ and C22^ - C24^ [upper abdominal noncolorectal gastrointestinal cancers], ICD-10 C50^ [breast cancer], ICD-10 C54^ [uterine cancer], ICD-10 C56^ [ovarian cancer], ICD-10 C65^ - C67^ [urothelial cancer] and ICD-10 C71^ [brain malignancies]). Among persons affected by each, we computed the mean age at diagnosis and the mean interval between the date of diagnosis and the index date. Rates were compared by *P*-values using a Poisson distribution assumption.

We described the colonoscopy on the index date from the OHIP database. We distinguished non-hospital facility colonoscopy from hospital outpatient colonoscopy by the presence of the OHIP feecode E749 indicating a non-hospital facility (11). We categorized the endoscopist by the specialty suffix on the physician billing number as gastroenterologist, general surgeon, internist versus other. We categorized procedures performed during the colonoscopy in the following hierarchy: (1) OHIP feecode E685 only (>= 3 cm sessile polypectomy), (2) OHIP feecode E685 plus Z571 (>= 3 cm sessile polypectomy plus removal of smaller polyp(s), (4) Z571 without E685 (removal of only smaller polyps), (5) E717 biopsy only or (6) none of the above). We also computed the percent of persons with >=1 polypectomy billing claim (1) on or within 6 months following the index date, and (2) > 6 months following the index date to the end of follow-up.

From the Registered Persons Database, we calculated for each person the follow-up time from the index date to June 30, 2020, the date of last follow-up, or the date of death, whichever came first, and then identified all records of colonoscopy with any billing fee code, following the index date to the date of last contact or June 30, 2020, whichever came first. We determined the percentage of persons up-to-date with colonoscopy to the end of each of 7 years following the index date.

From the Registered Persons Database, we calculated the follow-up time for each person from the index date to June 30, 2019, the date of last follow-up, or the date of death, whichever came first, and then identified all records of CRC in the OCR with diagnosis dates from the index date to the date of last contact or June 30, 2019, the most recent date upon which OCR records are reliably complete, whichever came first. We calculated the number of CRC diagnoses per 100,000 person-years, stratified by age and sex. To account for all CRC diagnoses on the index date as well as all metachronous CRC diagnoses for the same person, we plotted the Mean Cumulative Function for all diagnoses of CRC, stratified by colonoscopy billing claim feecode, sex, and age <40 years versus 40 to 49 years (12–14). This is a nonparametric technique used to estimate the average cumulative number of CRC diagnoses over time. To approximate an estimate of the average annual rate of CRC diagnoses, the number of CRC diagnoses was divided by the person-years of follow-up then divided by the mean years of follow-up, stratified by Z494 versus Z499, age <40 versus 40 to 49 years, and by sex.

We identified all records of potentially associated noncolorectal malignancies between the index date and the date of last contact or June 30, 2019, whichever came first. Among males, we calculated the number of diagnoses, from the index date to the date of last contact or June 30, 2019, per 100,000 person-years, stratified by billing claim feecode. Among females, we calculated the number of diagnoses per 100,000 person-years, with and without inclusion of cancers of the breast, uterus, and ovary. To approximate an estimate of the average annual rate of diagnosis of potentially associated noncolorectal malignancies, the number of diagnoses was divided by the person-years of follow-up then divided by the mean years of follow-up, stratified billing claim feecode, age and sex (for those malignancies occurring in both sexes).

## RESULTS

We identified one or more billing claims Z494 for screening colonoscopy among those with hereditary CRC syndromes among 14,846 persons <= 49 years of age between 2013 and 2017; 49,505 persons <= 49 years of age had one or more billing claims for Z499 (screening colonoscopy for first-degree relatives of affected persons) (Table 1). The majority of those with Z494 were < 40 years of age on the index date (7745 [52.2%]) and the mean age in this age group on the index date was 30.48 years (SD 6.18 years; median 31 years, interquartile range 26 to 36 years). Overall, for those with Z494 and Z499, the mean duration of time observed prior to the index date ranged from 18.64 to 20.38 years among those <40 years on the index date, and ranged from 22.90 to 23.63 years among those 40 to 49 years of age.

**Table 1.** Baseline characteristics

	Z494	Z499
	Screening colonoscopy for persons with hereditary CRC syndromes	Screening colonoscopy for first-degree relatives of persons affected by colorectal neoplasia
*N* overall	14,846	49,505
Females	7745 (52.2%)	27,634 (55.8%)
Males	7101 (47.8%)	21,871 (44.2%)
Age		
<40 years	7494 (50.5%)	7891 (15.9%)
Mean (SD) years	30.48 years (6.18 years)	34.08 years (4.83 years)
Median (IQR) years	31 years (26–36 years)	35 years (32–38 years)
40–49 years	7352 (49.5%)	41,614 (84.1%)
Mean (SD) years	44.93 years (2.89 years)	45.09 years (2.92 years)
Median (IQR) years	45 years (42–48 years)	45 years (43–48 years)
Year of index date		
2013	3721 (25.1%)	10,206 (20.6%)
2014	3301 (22.2%)	10,108 (20.4%)
2015	3001 (20.2%)	10,625 (21.5%)
2016	2468 (16.6%)	9716 (19.6%)
2017	2355 (15.9%)	8850 (17.9%)
Persons aged < 40 years up-to-date with biennial colonoscopy		
At 2 years prior to index	1443 (19.3%)	100 (1.3%)
At 4 years prior to index	1550 (20.7%)	228 (2.9%)
At 6 years prior to index	1315 (17.0%)	614 (7.8%)
At 8 years prior to index	992 (13.3%)	220 (2.8%)
At 10 years prior to index	757 (10.1%)	138 (1.8%)
Persons aged 40–49 years up-to-date with biennial colonoscopy		
At 2 years prior to index	1107 (15.1%)	202 (0.5%)
At 4 years prior to index	1605 (21.8%)	1203 (2.9%)
At 6 years prior to index	1605 (21.8%)	6041 (14.5%)
At 8 years prior to index	1186 (16.1%)	1777 (4.3%)
At 10 years prior to index	1007 (13.7%)	1162 (2.8%)
Persons aged <40 years with polypectomy		
<= 5 years prior to index date	741/7494 (9.90%)	184/7891 (2.33%)
> 5 years to 10 years prior to index date	302/7494 (4.00%)	155/7891 (1.96%)
Persons aged 40–49 years with polypectomy		
<= 5 years prior to index date	944/7352 (12.84%)	1092/41,614 (2.62%)
> 5 years to 10 years prior to index date	498/7352 (6.77%)	1590/41,614 (3.82%)

Prior to the index date, only a few were up-to-date with biennial screening colonoscopy (Table 1). Prior to the index date > 215/14,846 (1.5%) of those with Z494 had one or more potentially associated noncolorectal malignancies (Table 2, the exact count cannot be presented because of suppressed small cell counts for urothelial malignancies), as did > 421/49,505 (0.9%) of those with Z499. The rates of previously diagnosed potentially associated noncolorectal malignacies are higher among those with Z494 compared to those with Z499 for upper abdominal noncolorectal gastrointestinal and biliary cancers (*P* < 0.0001), uterine malignancies (*P* < 0.0001) and ovarian malignancies (*P* = 0.0006), but not for female breast cancer (*P* = 0.1) or brain malignancies (*P* = 0.3).

**Table 2.** Potentially associated noncolorectal malignancies prior to index date

	Screening colonoscopy for persons with hereditary CRC syndromes Z494	Screening colonoscopy for first-degree relatives of persons affected by colorectal neoplasia Z499	*p* Z494 vs. Z499
Upper abdominal GI cancer prior to index date			*P* < 0.0001
*N* of persons with >= 1 diagnosis	28/14,846 (0.2%)	12/49,505 (0.02%)	
*N* of diagnoses	29	13	
Age at earliest diagnosis date			
Mean (SD)	36.07 years (SD 12.35 years)	41.83 years (SD 4.09 years)	
Median (IQR)	40 years (IQR 33–45 years)	42 years (IQR 38–45 years)	
Interval from earliest diagnosis date to index date			
Mean (SD)	4.34 years (SD 3.10 years)	3.79 years (SD 3.07 years)	
Median (IQR)	2.39 years (IQR 0.76–5.08 years)	2.40 years (IQR 0.12–7.69 years)	
Uterine malignancy prior to index date			*P* < 0.0001
*N*	49/7745 (0.6%)	29/27,633 (0.01%)	
Age at earliest diagnosis date			
Mean (SD)	39.69 years (SD 5.25 years)	42.10 years (SD 3.87 years)	
Median (IQR)	40 years (IQR 37–42 years)	42 years (IQR 41–45 years)	
Interval from earliest diagnosis date to index date			
Mean (SD)	3.50 years (SD 3.10 years)	3.80 years (SD 3.07 years)	
Median (IQR)	2.39 years (IQR 1.30–5.09 years)	2.43 years (IQR 1.16–6.11 years)	
Ovarian malignancy prior to index date *P* = 0.4			
*N*	22/7745 (0.3%)	36/27,633 (0.013%)	
Age at earliest diagnosis date			*P* = 0.006
Mean (SD)	36.73 years (SD 8.87 years)	38.61 years (SD 7.62 years)	
Median (IQR)	39 years (IQR 34–42 years)	41 years (IQR 34–45 years)	
Interval from earliest diagnosis date to index date			
Mean (SD)	4.96 years (SD 5.53 years)	5.56 years (SD 5.40 years)	
Median (IQR)	2.44 years (IQR 1.65–6.25 years)	3.65 years (IQR 1.68–7.44 years)	
Female breast cancer prior to index date *P* < 0.0001			
*N* of person with >= 1 diagnosis	105/7745 (1.4%)	319 (1.2%)	*P* = 0.1
*N* of diagnoses	111	331	
Age at earliest diagnosis date			
Mean (SD)	38.23 years (SD 5.62 years)	41.04 years (SD 5.25 years)	
Median (IQR)	39 years (IQR 34–43 years)	42 years (IQR 38–48 years)	
Interval from earliest diagnosis date to index date			
Mean (SD)	4.96 years (SD 4.34 years)	4.82 years (SD 4.55 years)	
Median (IQR)	3.37 years (IQR 1.76–7.19 years)	3.03 years (IQR 1.47–6.89 years)	
Urothelial carcinoma prior to index date *P* = 0.45			
*N*	12		Not applicable
Age at earliest diagnosis date			
Mean (SD)	33.25 years (SD 6.20 years)		
Median (IQR)	32.08 years (IQR 30.33–37.58 years)		
Interval from earliest diagnosis date to index date			
Mean (SD)	9.78 years (SD 6.10 years)		
Median (IQR)	6.85 years (IQR 4.58–15.49 years)		
Brain malignancy prior to index date			*P* = 0.3
*N*	11/7745 (0.1%)	25/27,633 (0.01%)	
Age at earliest diagnosis date			
Mean (SD)	27.36 years (SD 11.32 years)	33.00 years (SD 11.99 years)	
Median (IQR)	29 years (IQR 23–36 years)	35 years (IQR 29–40 years)	
Interval from earliest diagnosis date to index date			
Mean (SD)	7.94 years (SD 9.01 years)	9.33 years (SD 9.98 years)	
Median (IQR)	4.11 years (IQR 1.24–15.33 years)	5.80 years (IQR 3.05–14.20 years)	

Descriptors of the colonoscopy on the index date differed between those with Z494 and those with Z499 (Table 3). For those with Z494, 8,956 (60.4%) the colonoscopy was performed by a gastroenterologist, and 12,727 (85.7%) underwent outpatient hospital-based colonoscopy as opposed to having their procedure in a non-hospital facility, compared to those with Z499, among whom gastroenterologists performed only 17,852 (36.1%) and 29,267 (59.1%) underwent outpatient hospital-based colonoscopy. At the index colonoscopy, 63 (0.4%) of those with Z494 underwent polypectomy for a >= 3 cm sessile lesion, with or without additional removal of smaller polyps, compared to 143 (0.3%) of those with Z499. Overall, a smaller percent of the Z494 cohort had no additional procedure (e.g., biopsy, polypectomy) during the colonoscopy 7561 (50.9%), compared to 32,494 (65.6%) of the Z499 cohort. For several years following the index date, the percent up-to-date with biennial colonoscopy was >= 80%, however by the seventh year, utilization was between 40% and 50% (Table 4). On or within six months following the index date, more persons with Z499 underwent polypectomy, however beyond 6 months following the index date, more persons with Z494 underwent polypectomy.

**Table 3.** Age-stratified colorectal cancer diagnoses and potentially associated age-stratified other malignant diagnoses from the index date to the end of follow-up

CRC on or following the index date	Screening colonoscopy for persons with hereditary CRC syndromes Z494	Screening colonoscopy for first-degree relatives of persons affected by colorectal neoplasia Z499
Females		
<40 years	11/15,752.67 person-years	7/17,585.99 person-years
	16.4 per 100,000 per year	9.8 per 100,000 per year
40–49 years	20/17,134.48 person-years	55/95,074.98 person-years
	27.5 per 100,000 per year	14.2 per 100,000 per year
All Females	31/32,887.15 person-years	62/112,660.97 person-years
	22.2 per 100,000 per year	13.5 per 100,000 per year
Males		
< 40 years	19/15,386.68 person-years	5/13,857.81 person-years
	27.8 per 100,000 per year	9.1 per 100,000 per year
40–49 years	28/14,501.09 person-years	51/72,889.58 person-years
	45.6 per 100,000 per year	17.6 per 100,000 per year
All Males	47/29,887.77 person-years	56/86,747.38 person-years
	38.6 per 100,000 per year	16.3 per 100,000 per year
Potentially associated noncolorectal cancers on or following the index date		
Females		
All	75/32,887.15 person-years	284/112,660.97 person-years
	53.7 per 100,000 per year	61.8 per 100,000 per year
Breast cancer	46/32,887.15 person-years	202/112,660.97 person-years
	32.9 per 100,000 per year	43.5 per 100,000 per year
Endometrial cancer	13/32,887.15 person-years	27/112,660.97 person-years
	9.3 per 100,000 per year	5.9 per 100,000 per year
Ovarian cancer	7/32887.15 person-years	16/112660.97
	5 per 100,000 per year	3.5 per 100,000 per year
Other cancers	11/32887.15 person-years	39/112,660.97
	7.9 per 100,000 per year	8.5 per 100,000 per year
Males		
All	22/29,887.77 person-years	32/86,747.38 person-years
	17.5 per 100,000 per year	9.3 per 100,000 per year

**Table 4.** Colonoscopy on the index date and ‘up-to-date’ status with biennial colonoscopy

	Screening colonoscopy for persons with hereditary CRC syndromes Z494 *n* = 14,846	Screening colonoscopy for first-degree relatives of persons affected by colorectal neoplasia Z499 *n* = 49,505
Index date colonoscopy		
Endoscopist specialty		
Gastroenterology	8965 (60.4%)	17,852 (36.1%)
General Surgery	2638 (17.8%)	25,208 (50.9%)
Internal Medicine	3002 (20.2%)	5681 (11.5%)
Other	241 (1.6%)	764 (1.5%)
Index date colonoscopy setting		
Outpatient hospital	12,727 (85.7%)	29,267 (59.1%)
Non-hospital facility	2119 (14.3%)	20,238 (40.9%)
Diagnosis of CRC on index date ± 7 days		
Females	9	33
Males	10	40
Index date colonoscopy procedures (mutually exclusive)		
>= 3 cm sessile polypectomy only	30 (0.2%)	59 (0.1%)
>= 3 cm sessile polypectomy plus removal of smaller polyps	33 (0.2%)	84 (0.2%)
Removal of smaller polyps only	2367 (15.9%)	13,936 (28.2%)
Biopsy only	4855 (32.7%)	2932 (5.9%)
None of the above	7561 (50.9%)	32,494 (65.6%)
Up-to-date with biennial colonoscopy, persons aged <40 years on index date		
At 3 years after the index date	98.7%	97.3%
At 4 years after the index date	97.1%	94.1%
At 5 years after the index date	85.0%	78.3%
At 6 years after the index date	67.3%	60.8%
At 7 years after the index date	48.4%	42.8%
Up-to-date with biennial colonoscopy, persons aged 40–49 years on index date		
At 3 years after the index date	99.0%	98.5%
At 4 years after the index date	97.5%	96.1%
At 5 years after the index date	86.9%	82.4%
At 6 years after the index date	71.5%	63.1%
At 7 years after the index date	52.6%	42.9%
Persons aged < 40 with polypectomy		
On or within 6 months following index date	1011/7494 (13.49%)	1714/7891 (21.72%)
> 6 months following index date	1153/7494 (15.68%)	664/7891 (8.41%)
Persons aged 40–49 years with polypectomy		
On or within 6 months following index date	1464/7352 (19.91%)	12,496/41,614 (30.03%)
>6 months following index date	1580/7352 (21.49%)	4872/41,614 (11.71%)

Among those at age < 40 years on the index date, the mean duration of follow-up from the index date to the date of last contact or June 30, 2019 in the OCR database ranged from 3.88 to 4.19 years stratified by billing claim feecode and sex; and their follow-up time from the index date to the date of last contact or June 30, 2020 in the OHIP database ranged from 4.33 to 4.86 years. Among those at age 40 to 49 years on the index date, the mean duration of follow-up from the index date to the date of last contact or June 30, 2019 in the OCR database ranged from 3.98 to 4.30 years, and from the index date to the date of last contact or June 30, 2020 in the OHIP database ranged from 4.50 to 4.96 years.

**Figure 1.**
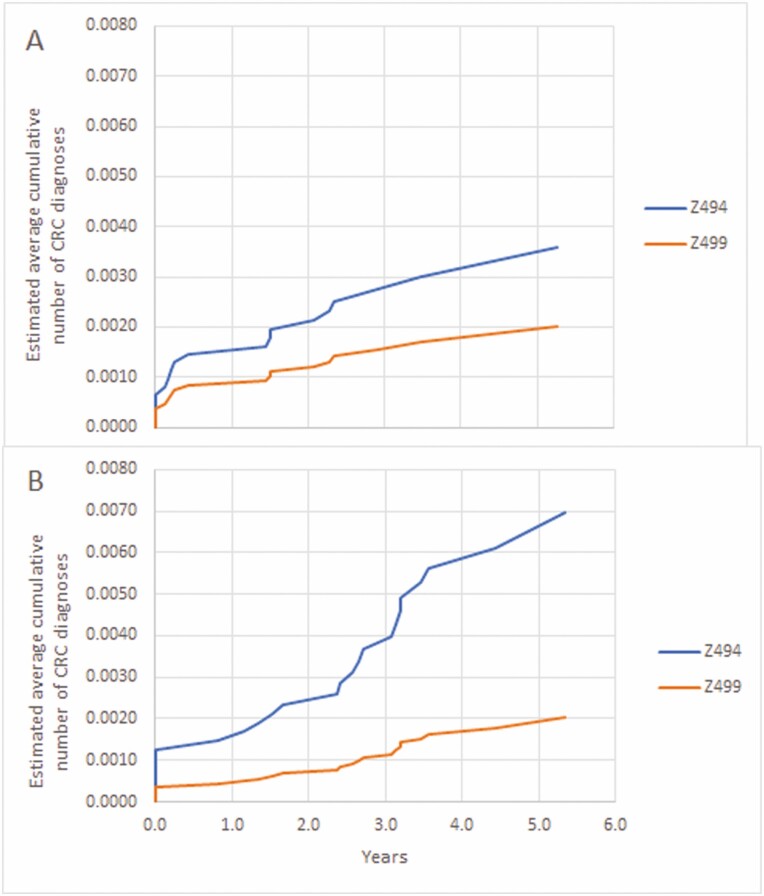
Mean cumulative function of colorectal cancer diagnoses by colonoscopy feecode. (A) Females <= 39 years of age on index date. (B) Males <= 39 years of age on index date.

**Figure 2.**
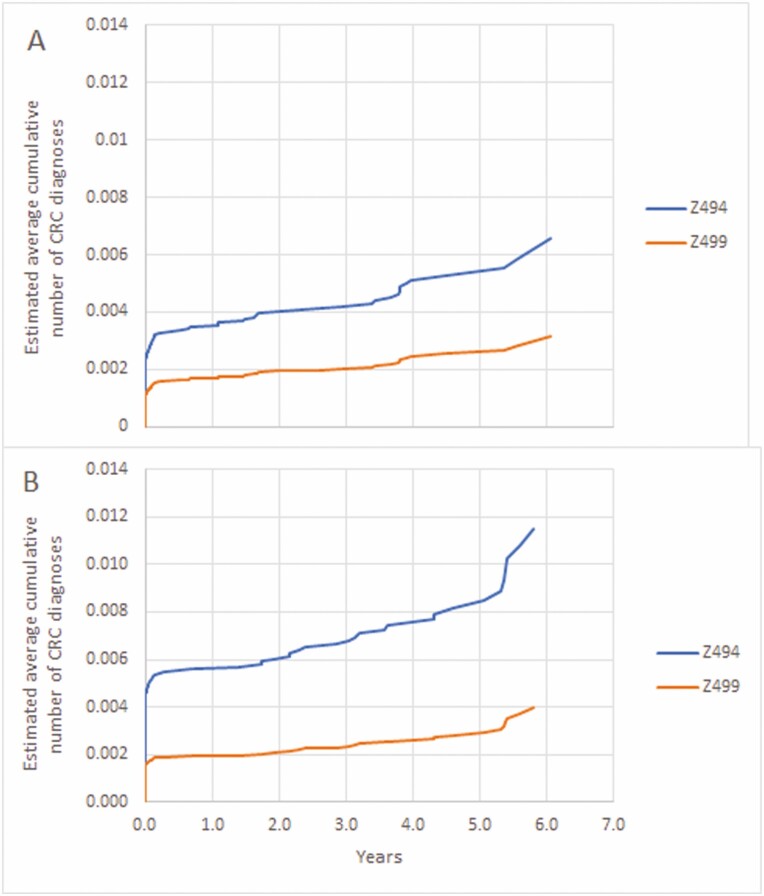
Mean cumulative function of colorectal cancer diagnoses by colonoscopy feecode. (A) Females 40 to 49 years of age on index date. (B) Males 40 to 49 years of age on index date.

From the index date to the date of last contact or June 30, 2019, whichever came first, among those with Z494, there were 78 CRC diagnoses among 59 persons; only 19/78 (24.4%) were on the index date or within 7 days. The stage of the first CRC was Stage 1 for 19 (32.2%), Stage 4 for 10 (17.0%) and missing for 9 (15.3%). Among those with Z499, there were 118 CRC diagnoses among 107 persons; 73/118 (61.9%) were on the index date or within 7 days. The stage of the first CRC was Stage 1 for 48 (44.9%), Stage 4 for 7 (6.5%) and missing for 19 (17.8%). Among females with Z494 the average annual rate of CRC diagnosis was 22.2 per 100,000, compared to 13.5 per 100,000 with Z499. The Mean Cumulative Function of CRC diagnoses among females stratified by colonoscopy feecode and age on index date is plotted in Figure 1A and B. Among males the average annual rate of CRC diagnoses was 38.6 per 100,000 persons with Z494 compared to 16.3 per 100,000 persons with Z499. The Mean Cumulative Function of CRC diagnoses among males stratified by colonoscopy feecode and age on index date is plotted in Figure 2A and B.

Between the index date and the date of last contact or June 30, 2019, whichever came first, the average annual rate of female breast cancer was 32.9 per 100,000 women, of uterine cancer was 9.3 per 100,000 women, and ovarian cancer was 5.0 per 100,000 among females with a Z494 billing claim, compared to the average annual rate of 43.5 female breast cancers per 100,000 women, 5.9 uterine cancers per 100,000 women, and 3.5 ovarian cancers per 100,000 women with a Z499 billing claim. For upper abdominal noncolorectal cancers, urothelial cancers, and brain malignancies combined, the average annual rate was 17.5 diagnoses per 100,000 per year among males and 9.3 diagnoses per 100,000 per year among females with a Z494 billing claim, compared to 7.9 cases and 8.5 cases per 100,000 per year respectively with a Z499 billing claim.

## Discussion

The average annual rate of CRC diagnoses from the index date to the date of last contact or June 30, 2019, whichever came first, was substantially higher among those with a Z494 billing claim compared to those with a Z499 billing claim, and compared to age-stratified unsmoothed overall national rates for the general population reported on the Statistics Canada website for 2013 to 2017 (15). This is consistent with a hypothesis that the Z494 subcohort is enriched with persons at elevated CRC risk. The significantly higher past history of upper abdominal noncolorectal gastrointestinal malignancies, uterine and ovarian care prior to the index date suggests that some persons with one or more Z494 billing claims do have a familial colorectal cancer syndrome.

The CRC rates observed among those with a Z499 billing claim were similar to, or lower than those reported by Statistics Canada. However, a larger percent of those persons had one or more polypectomies on or during the 6 months following the index date compared to those with Z494; beyond 6 months following the index date to the end of follow-up, this was reversed.

Between the index date and last follow-up, the average annual rates of uterine cancer and ovarian cancer were somewhat higher among those with a Z494 billing claim compared to those with a Z499 claim, but were not higher than the Statistics Canada rates (15). The average annual rate of female breast cancer was higher among those with a Z499 billing claim, possibly because of the higher proportion of women age 40 to 49 years compared to those with Z494. The average annual rates of potentially associated noncolorectal malignancies appeared to be substantially higher among males with Z494 compared to Z499, however, the absolute summed counts were low, and the rates could not be disaggregated for comparison to those of Statistics Canada because of suppressed small cell counts. Excluding female breast cancer, uterine and ovarian cancer, the aggregated average annual rates among females were similar between the Z494 and Z499 cohorts. Among the Z494 subcohort, the percent up-to-date with biennial colonoscopy was fairly strong up to the fifth year after the index date, after which it declined to about 50%, which would not be concordant with guidelines (2) to the extent that these persons actually have a familial CRC syndrome. The Danish HNPCC register has reported a mean interval of 2.1 years between colonoscopies among persons with Lynch Syndrome, however, with a range of 0.5 to 13.3 years) (16,17). While there are high quality, research-based familial colorectal cancer registries in Canada (18,19), including Ontario (20–23), Vasen et al. (24) recommend a jurisdiction-wide, population-based registry which would additionally facilitate recall for periodic colonoscopy.

There are several limitations to this study. Although we have population-wide records for all colonoscopies and all cancers in Ontario, we have no information on the presence of any mutation predisposing to colorectal cancer for any individual or family in Ontario, nor do we have any information about the family history of colorectal neoplasia for any individual. Engel et al. (17) found no difference in outcomes among jurisdictions with annual, biennial or triennial high-risk screening colonoscopy; because of the brief follow-up after the index date, we cannot assess any relationship between the inter-colonoscopy interval and outcomes. Although Sjostrom et al. (25) found a protective benefit against colorectal cancer incidence among families with hereditary syndromes or other family history of colorectal cancer from frequent colonoscopy in the community setting, our follow-up is brief and we cannot identify persons at increased risk of CRC who do not have any billing claims for Z494 or Z499. The appropriateness of the use of each of the ten colonoscopy billing claim feecodes has not been audited. We are unable to determine if those with a Z499 billing claim <= 49 years of age are a high-risk population of first-degree relatives because of our short follow-up after the index date; much longer follow-up after the index date would be required to identify the presence or absence of increased risk of CRC consistent with first-degree relatives of affected cases. Furthermore, their exposure to colonoscopic polypectomy, preventing CRC, may reduce the possibility of measuring an increased risk. Finally, we are unable to determine from our data sources the proportion of those who become non-compliant with biennial colonoscopy who actually have a familial colorectal cancer syndrome.

## CONCLUSION

Colorectal cancer was more frequent following billing claims for high-risk screening colonoscopy for familial syndromes, as were noncolorectal malignancies potentially associated with these syndromes. There may be a need to establish a systematic jurisdiction-wide registry to facilitate recall to periodic colonoscopy.
